# Synapse‐enriched miRNA expression in Alzheimer’s disease cortex tissue

**DOI:** 10.1002/alz.090741

**Published:** 2025-01-03

**Authors:** Laia Lidón Gil, Raúl Núñez‐Llaves, Sara Serrano‐Requena, Danna Perlaza, Alba Cervantes‐González, Natalia Valle Tamayo, Esther Álvarez‐Sánchez, Érika Sánchez‐Aced, Sonia Sirisi Dolcet, Oriol Dols‐Icardo, Alberto Lleo, Rocío Pérez‐González, Olivia Belbin

**Affiliations:** ^1^ Sant Pau Memory Unit, Hospital de la Santa Creu i Sant Pau ‐ Biomedical Research Institute Sant Pau ‐ Universitat Autònoma de Barcelona, Barcelona Spain; ^2^ Center for Biomedical Investigation Network for Neurodegenerative Diseases (CIBERNED), Madrid Spain; ^3^ Ace Alzheimer Center, Barcelona Spain; ^4^ Memory Unit, Department of Neurology, Institut de Recerca Sant Pau ‐ Hospital de Sant Pau, Universitat Autònoma de Barcelona, Barcelona, Spain. Centro de Investigación Biomédica en Red en Enfermedades Neurodegenerativas (CIBERNED), Madrid, Spain, Barcelona, Barcelona Spain; ^5^ Instituto de Investigación Sanitaria y Biomédica de Alicante (ISABIAL), Instituto de Neurociencias, Universidad Miguel Hernández‐CSIC, Alicante Spain

## Abstract

**Background:**

Synapse degeneration is one of the earliest changes in Alzheimer’s disease (AD) and is the major neuropathologic correlate of cognitive impairment. The aim of this study was to characterize microRNA (miRNA) dysregulation at AD synapses post‐mortem brain tissue and explore the specificity for AD.

**Method:**

We prepared synaptosomes (SYN) by serial ultracentrifugation of 10 AD (mean age = 77.4±13.3) and 10 control (CN; mean age = 72.3±18.8) temporal cortex samples over a sucrose gradient and saved the homogenates (H). We extracted and quantified miRNA from SYN and H fractions by TaqMan Advanced miRNA Assay‐Openarray system. We calculated deltaCt and performed linear regression to identify differentially expressed (DE) miRNA across AD and CN SYN, adjusting for multiple testing using Benjamini‐Hochberg method (α<0.05). We identified gene targets of the DE miRNA using two predictive databases (TargetScan and miRDB) and performed pathway analysis using the Panther database. We selected miRNA candidates that are DE in AD synapses and performed a follow‐up study in 4 different brain regions of 10 CN (mean age = 80.7±12.9), 10 AD (mean age = 77.4±13.3) and 10 non‐AD tauopathy (mean age = 70.6±6.4) cases to determine the regional distribution and the AD specificity.

**Result:**

Synapse fractions were isolated demonstrating a 3.2‐fold enrichment in the synaptic protein VAMP‐2 (p<0.001) compared to homogenates. We detected 411 miRNA in H and 432 in SYN of the total in the panel with 89% overlap between AD and CN in SYN. Three miRNA from the same family (miR‐132‐3p, miR‐132‐5p and miR‐212‐3p) were under‐expressed and 2 miRNA (miR‐181a‐3p and miR‐1260a) were over‐expressed in AD compared to CN SYN. The gene targets of these miRNA were overrepresented in pathways regulating synaptic and mitochondrial function. In addition, targets of miR‐132 also were overrepresented in pathways related to tau pathology, Aβ production, interleukin production, apoptosis and oxidative stress. We will describe the regional distribution in AD brains post‐mortem and the specificity of the changes to AD by comparing to non‐pathological controls and non‐AD tauopathies.

**Conclusion:**

miRNA that are dysregulated in AD synapses target genes that are involved in pathways related to AD pathogenesis. Future studies will focus on the use of these miRNA as therapeutic targets.

